# Direct effects of warming increase woody plant abundance in a subarctic wetland

**DOI:** 10.1002/ece3.3902

**Published:** 2018-02-11

**Authors:** Lindsay G. Carlson, Karen H. Beard, Peter B. Adler

**Affiliations:** ^1^ Department of Wildland Resources and the Ecology Center Utah State University Logan UT USA

**Keywords:** competition coefficient, goose herbivory, indirect effects, plant–climate interactions, shrub expansion, species interactions, subarctic

## Abstract

Both the direct effects of warming on a species’ vital rates and indirect effects of warming caused by interactions with neighboring species can influence plant populations. Furthermore, herbivory mediates the effects of warming on plant community composition in many systems. Thus, determining the importance of direct and indirect effects of warming, while considering the role of herbivory, can help predict long‐term plant community dynamics. We conducted a field experiment in the coastal wetlands of western Alaska to investigate how warming and herbivory influence the interactions and abundances of two common plant species, a sedge, *Carex ramenskii*, and a dwarf shrub, *Salix ovalifolia*. We used results from the experiment to model the equilibrium abundances of the species under different warming and grazing scenarios and to determine the contribution of direct and indirect effects to predict population changes. Consistent with the current composition of the landscape, model predictions suggest that *Carex* is more abundant than *Salix* under ambient temperatures with grazing (53% and 27% cover, respectively). However, with warming and grazing, *Salix* becomes more abundant than *Carex* (57% and 41% cover, respectively), reflecting both a negative response of *Carex* and a positive response of *Salix* to warming. While grazing reduced the cover of both species, herbivory did not prevent a shift in dominance from sedges to the dwarf shrub. Direct effects of climate change explained about 97% of the total predicted change in species cover, whereas indirect effects explained only 3% of the predicted change. Thus, indirect effects, mediated by interactions between *Carex* and *Salix,* were negligible, likely due to use of different niches and weak interspecific interactions. Results suggest that a 2°C increase could cause a shift in dominance from sedges to woody plants on the coast of western Alaska over decadal timescales, and this shift was largely a result of the direct effects of warming. Models predict this shift with or without goose herbivory. Our results are consistent with other studies showing an increase in woody plant abundance in the Arctic and suggest that shifts in plant–plant interactions are not driving this change.

## 1 INTRODUCTION

Climate change can influence plant communities through both direct and indirect effects. Direct effects occur when warming alters plant populations through changes in a focal species’ own vital rates (Adler, Leiker, & Levine, 2009). Indirect effects occur when warming alters the vital rates and abundances of neighboring species, which in turn affects the fitness of the focal species (Adler et al. 2012; Adler et al., 2009; Gilman, Urban, Tewksbury, Gilchrist, & Holt, 2010). While the relative importance of these two mechanisms is still being explored, theory and some empirical evidence show that direct effects will dominate in communities where plant species have little niche overlap (Chu et al., 2016; Kleinhesselink & Adler, 2015). In contrast, where plants occupy the same niche, indirect effects appear more important and can even override direct effects (Gilman et al., 2010; Klanderud, 2005; Suttle, Thomsen, & Power, 2007; Tylianakis, Didham, Bascompte, & Wardle, 2008). In communities with strong indirect effects, climate change projections that do not account for these interactions will not adequately predict future abundances of important species (Levine, Adler, & HilleRisLambers, 2008; Mod, le Roux, Guisan, & Luoto, 2015; Suttle et al., 2007; Tylianakis et al., 2008).

Over the past 150 years, northern latitudes have experienced dramatic increases in temperature, two to three times greater than the global mean surface temperature rise of 0.4°C (IPCC 2014). We might expect direct effects of climate to be more important than indirect effects in northern systems because of the lack of strong competitive interactions between species in severe environments, such as northern or alpine ecosystems (Callaway et al., 2002; Cavieres et al., 2014). However, the importance of indirect effects of climate change has not been well studied in Arctic systems, and recent studies suggest interspecific interactions could become increasingly important in these systems with warming (Klanderud, 2005; Klanderud, Vandvik, & Goldberg, 2015). Warmer temperatures have been linked to the range expansion and increasing abundance of shrubs and woody plants in Arctic tundra and alpine ecosystems (Elmendorf et al., 2012; Myers‐Smith et al., 2011; Sturm et al., 2001; Tape, Sturm, & Racine, 2006), but it is unknown whether direct or indirect effects are driving this change.

While warming has been shown to influence community composition in northern latitudes, herbivory has been found to counteract the effects of warming in some systems by maintaining plant species composition and preventing shrub expansion (Christie et al., 2015; Kaarlejärvi, Hoset, & Olofsson, 2015; Olofsson et al., 2009; Post & Pedersen, 2008). More so than mammalian herbivores, migratory geese rely on nutrient‐rich herbaceous vegetation, such as sedges, in their Arctic and subarctic breeding areas (Doiron, Gauthier, & Levésque, 2015; Post et al., 2009; Sedinger & Raveling, 1984). Because migratory geese are abundant during the short growing season, they have the potential to transform vegetation at the landscape scale and increase the nutrient content of grazed plants (Cargill & Jefferies, 1984; Person, Babcock, & Ruess, 1998; Sedinger et al., 2016). However, if climate change favors woody plants over preferred nutrient‐rich sedges in their breeding ground, it could reduce the amount of forage available for some goose herbivores. Thus, it is important to consider whether current levels of grazing pressure support sedge growth and through its competitive ability with shrubs, prevent shrub expansion in the light of warming.

The goal of our research was to disentangle the effects of climate warming, herbivory, and plant–plant interactions on a subarctic coastal wetland community. We had three main objectives. First, we conducted an experiment, using a response surface design, to determine how the abundances of two dominant species, *Carex ramenskii* (sedge) and *Salix ovalifolia* (dwarf shrub), change under warmed and grazed conditions. Second, we used our experimental data to parameterize competition models to predict the equilibrium abundances of these species under warmed and grazed conditions and to determine whether herbivory mediates the effects of warming in the long term. Finally, we determined the relative importance of direct versus indirect effects of warming, with or without grazing, on plant species abundance.

## 2 METHODS

### 2.1 Study site

Our research was conducted on the Tutakoke River in the central portion of the coastal Yukon–Kuskokwim (Y‐K) Delta in western Alaska (61°15′N, 165°30′W; elevation 3 m). The Y‐K Delta is 75,000 km^2^ of subarctic wetland and tundra between the Yukon and Kuskokwim Rivers, and along the coast of the Bering Sea. Climate in the region is maritime, with mean monthly temperature ranging from −14.1°C in midwinter to 13.3°C in midsummer with a growing season from late May through late August (Terenzi, Jorgenson, & Ely, 2014). Mean annual rainfall is 41.1 cm and snowfall is 157 cm (Terenzi et al., 2014).

The Y‐K Delta is an important breeding area for migratory birds (Baldassarre, 2014). Our site provides primary nesting and brood‐rearing habitat mainly for a colony of Pacific black brant (*Branta bernicla nigricans*) but cackling geese (*B. hutchinsii minima*) are also common, and emperor geese (*Chen canagica*) and greater white‐fronted geese (*Anser albifrons*) utilize the area in small numbers during the early season (Ruess, Uliassi, Mulder, & Person, 1997). In recent decades, the number of cackling geese and greater white‐fronted geese breeding in the Y‐K Delta has increased (Fischer & Stehn, 2014; Ruess et al., 1997). Alaskan moose (*Alces alces gigas*) are common in inland areas and also have been increasing their range and abundance in coastal areas in recent years (Tape, Gustine, Ruess, Adams, & Clark, 2016; J. Sedinger and J. Schmutz, personal communication).

Our experiment was conducted in a brackish wet sedge meadow on the active floodplain. The meadow is 10–20 cm higher than adjacent tidal channels; the soil is silty loam underlain with deposits of silts and sands and has neutral soil pH (Jorgenson, 2000). Soil moisture content typically exceeds 50% during the growing season (unpublished data). *Carex ramenskii*, a salt‐tolerant sedge, is the dominant species within 3 km of the coast (Jorgenson, 2000; Kincheloe & Stehn, 1991). *C. ramenskii* has a shorter, more nutritious growth form (often referred as *C. subspathacea* or grazing lawn), which is the preferred forage for black brant geese and goslings (Sedinger & Raveling, 1984).

At our study site, *C. ramenskii* is intermixed with the dwarf shrub, *Salix ovalifolia* (hereafter *Carex* and *Salix*). At the peak of the growing season in control plots in 2015, *Carex* cover was 55% ± 16 *SD*,* Salix* cover was 37% ± 12 *SD*, all other species made up <3% cover, and remaining cover was dead biomass or bare ground. While *Salix* is not the preferred forage of some geese species, like black brant, other species, such as cackling geese and greater white‐fronted geese, have less‐restrictive diets and moose may prefer it.

### 2.2 Experimental methods

To accomplish our first objective, to conduct an experiment using a response surface design to determine how the abundances of *Carex* and *Salix* change under warmed and grazed conditions, we conducted a two‐season field experiment during the spring and summer of 2015 and 2016. In May 2015, we established 80, 0.85‐m‐diameter circular plots. Within each plot, we established four circular (20‐cm diameter) subplots or “neighborhoods.” The four neighborhoods were randomly placed in nonoverlapping areas in the interior 0.8‐m diameter of the plot (to limit edge effects). The center of each neighborhood was marked so that the exact subplot could be remeasured. The data analyzed in this study is the percent cover of both species in each neighborhood subplot at the beginning and end of the experiment using the point‐intercept method. Initial cover was measured shortly after the removal treatments were completed by mid‐June 2015, before peak hatch (June 20), and after all vegetation was greened and leafy (Fischer & Stehn, 2014). Final cover was measured at the end of the growing season (mid‐August) in 2016.

To create the warming and grazing treatments, we had a factorial combination of two factors, warming (+/−) and grazing (+/−). Treatments were as follows: ambient temperature, grazed (hereafter, ambient, grazed); ambient temperature, ungrazed (hereafter, ambient, ungrazed); warmed, grazed; and warmed, ungrazed. We created warming treatments using fiberglass open‐top warming chambers (OTCs) following International Tundra Experiment specifications (as in Molau & Mølgaard, 1996). Thermochron iButtons in our plots showed that OTCs raised air temperature at the soil surface by on average 1.75°C over the growing season (mean minimum increase 1°C; mean maximum increase 5°C). OTCs were not left in place over the winter because the area floods frequently in the fall. We used OTCs because, unlike greenhouses, they minimally alter precipitation and gas exchange (Marion et al., 1997; Molau & Mølgaard, 1996). Because OTCs exclude herbivores, warming and natural grazing could not be simultaneous. Therefore, we exclosed all treatments from natural herbivory using OTCs on warmed plots and 1‐m tall, 2.54‐cm hexagonal mesh fencing on ambient temperature plots.

We simulated grazing treatments by manually clipping vegetation‐grazed plots on four occasions throughout the season. We based grazing treatments on black brant seasonal biomass offtake at the study site (Person et al., 1998). Both species received the same intensity of grazing with respect to its proportion of total cover. However, clipping was done differently for the species because of their different growth forms. *Carex* was clipped such that the tops of the tillers were removed; tiller basal stems and roots were not clipped or pulled in this treatment. For *Salix,* whole leaves were removed and occasionally the ends of runners were clipped where leaf biomass was insufficient to meet the target biomass. We normally distributed the amount of vegetation clipped across four dates 30 days after black brant peak hatch, when herbivory is greatest (Sedinger & Flint, 1991). To simulate fecal deposition, we added goose feces four times per season on the same dates as clipping to each plot receiving grazing based on nearby fecal deposition monitoring plots. In 2015, we added 1‐2‐1‐1 feces, and in 2016, we added 1‐2‐1‐0 feces during late June, early July, late July, and early August, respectively.

We did not simulate grubbing in addition to the aboveground tissue removal clipping treatment because the primary herbivores in our system, black brant and cackling geese, do not grub during the breeding season (Sedinger & Raveling, 1984). Other geese present, such as emperor geese and greater white‐fronted geese, do grub, but prefer to nest and raise broods further inland where upland and tundra vegetation communities are more prevalent; therefore, these species do not contribute substantial grazing pressure at our study site (Fischer & Stehn, 2014).

Nested within these four treatments, we also conducted vegetation removals to create a response surface design. There were four removal targets to create plots where 1) *Carex* was low but *Salix* was high (high = natural density), 2) *Salix* was low but *Carex* was high, 3) both were reduced, or 4) both were at natural density. We achieved this using the following removal targets: 95% removal of *Carex* and 0% removal of *Salix*, 95% removal of *Salix* and 0% removal of *Carex*, 50% removal of both *Carex* and *Salix*, and 0% removal of *Carex* nor *Salix* (Figure S1)*. *To be clear, our analysis ignores the categorical removal targets and just uses the continuous variation in initial (postremoval) cover. We implemented the removals by hand‐pulling plants each year in May. This removal method, unlike the clipping treatment simulating herbivory, did disturb belowground biomass. We pulled each plant by hand so that we would remove the basal stem and as much root biomass as possible, in the case of *Carex,* and the belowground portion of the runner, in the case of *Salix*. We recognize this method of removal may release nutrients, alter soil density and moisture, and affect neighboring plants, which is a limitation to our in situ study.

We assigned removal targets based on the initial percent cover of each species in the plot as quantified by the point‐intercept method. We repeated point‐intercept counts after removals to record postremoval percent cover (Veblen 2008). It was possible to have percent cover greater than 100% because our sampling method allowed for multiple hits per point‐intercept. Throughout the experiment, we continuously removed any non‐*Carex* or *Salix* species. To limit belowground interactions, we trenched around each 0.85‐diameter plot then inserted 0.8‐mm root barrier to 25 cm below the soil surface (as in Veblen 2008). The plastic barrier remained in place throughout the experiment. If anything, we expected such a barrier might harm *Salix* over *Carex* because *Salix* has a prostrate growth form, which utilizes horizontal runners for clonal reproduction.

### 2.3 Statistical methods

To accomplish our second objective, to predict how the equilibrium abundances of *Carex* and *Salix* change under warmed and grazed conditions, we first determined how warming and grazing affected the strength and direction of intra‐ and interspecific interactions between *Carex* and *Salix*. To do so, we fit data from our response surface removal experiment to seven candidate competition models (Table S1; Hart & Marshall, 2013; Inouye, 2001; Law & Watkinson, 1987; Levine & HilleRisLambers, 2009). We used nonlinear least squares to fit the experimental data (neighborhood cover) to the model and estimate parameter values. We fit models using the nls() function and the port algorithm in base R v. 3.3.2 (R Development Core Team 2014). We opted to fit models using nls() and exclude the random effect of neighborhood because when tested in exploratory models, random effects were small (orders of magnitude smaller than the residual). Using nonlinear least squares estimation, the best fit of the competition equation to the experimental data is obtained by minimizing the residual sums of squares to provide the least squares estimate of the parameters.

We included treatment as a four‐level categorical grouping factor that allows parameters to vary by treatment, so that we could simultaneously fit all of our experimental data for each species using a single model (Ritz & Streibig, 2008). The removal targets were not treated as categorical variables here; rather, the postremoval percent cover of the plots was created in a response surface design to vary the density of *Carex* and *Salix* independently such that competition models could be fitted explicitly. These density combinations can be thought of as a gradient of starting covers for each species to broaden the range of inference of our models to the bounds of the natural conditions.

Each model had three parameters for each species: λ, the density‐independent growth rate, α_*ii*_, the per capita (or per unit cover) effect of intraspecific neighbors, and α_*ij*_, the per capita effect of interspecific neighbors. By allowing these parameters to vary among each of the four treatments, the resulting models had 12 total parameters. For our first step in model selection, we used Akaike Information Criterion (AIC) to determine which candidate model best described our system (Table 1). The model that best described our data is a modified Ricker model (Ricker, 1954):

$$N_{c,t+1}=N_{c,t}(\lambda_{\left[\mathrm{tx}\right]}\exp\left(-{\alpha_{\mathrm{cc}}}_{\left[\mathrm{tx}\right]}\log\left(N_{c,t}\right)-{\alpha_{\mathrm{cs}}}_{\left[\mathrm{tx}\right]}\log\left(N_{s,t}\right)\right)$$

where *N*_*c,t*_ and *N*_*s,t*_ are the initial (postremoval) percent covers of *Carex* and *Salix*, respectively, and *N*_*c,t+1*_ is the final percent cover of *Carex* at the end of the second growing season. The subscript [*tx*] denotes where we allowed coefficients to vary between the four treatments. We repeated model fitting for *Salix* with the same notation:

$$N_{s,t+1}=N_{s,t}(\lambda_{\left[\mathrm{tx}\right]}\exp\left(-{\alpha_{\mathrm{ss}}}_{\left[\mathrm{tx}\right]}\log\left(N_{s,t}\right)-{\alpha_{\mathrm{sc}}}_{\left[\mathrm{tx}\right]}\log\left(N_{c,t}\right)\right).$$

**Table 1:** Results of Akaike Information Criterion (AIC) model selection, number of estimated parameters (*k*), difference in AIC_c_ between best model and model *i* (ΔAIC_c_), Akaike's weight which indicates weight of evidence in favor of model *i* (*w*_*i*_), negative log‐likelihood (−2lnl)*. *Candidate models excluded from this table were not able to be fit for both species due to convergence failure. The response variable for all models is *N*_*i,t+1*_, and model structure is *N*_*i,t+1*_ = *N*_*i,t*_ *f*(*X*_*t*_ *,Y*_*t*_)

Candidate models *f*(*X*_*t*_,*Y*_*t*_)	*k*	AIC_c_	ΔAIC_c_	*w*_i_	−2lnl
Carex					
$\lambda e^{-\alpha_{\mathrm{cc}}\ln\left(N_{c,t}\right)-\;\alpha_{\mathrm{cs}}\ln\left(\;N_{s,t}\right)}$	13	−162.15	0.00	1	−187.15
$\lambda e^{-\alpha_{\mathrm{cc}}N_{c,t}-\;\alpha_{\mathrm{cs}}\;N_{s,t}}$	13	−82.58	79.57	0	−108.58
$1+\lambda \left(1-\alpha_{\mathrm{cc}}N_{c,t}-\alpha_{\mathrm{cs}}N_{s,t}\right)$	13	−42.08	120.06	0	−68.08
Salix					
$\lambda e^{-\alpha_{\mathrm{ss}}\ln\left(N_{s,t}\right)-\;\alpha_{\mathrm{sc}}\ln\left(\;N_{c,t}\right)}$	13	−286.65	0.00	1	−312.65
$\lambda e^{-\alpha_{\mathrm{ss}}N_{s,t}-\;\alpha_{\mathrm{sc}}\;N_{c,t}}$	13	−259.79	26.87	0	−285.79
$1+\lambda \left(1-\alpha_{\mathrm{ss}}N_{s,t}-\alpha_{\mathrm{sc}}N_{c,t}\right)$	13	−237.04	49.62	0	−263.04

For the second step of our model selection procedure, we simplified the best model using likelihood ratio tests (LRT) to remove parameters that did not improve goodness of fit at confidence level of 0.10 (Hart & Marshall, 2013; Ritz & Streibig, 2008). We first determined whether a model that allows all parameters to vary by treatment is more favorable than a model that holds a particular parameter constant across treatments while allowing the other parameters to vary by treatment. We repeated this process across all three parameters for both species models. We also conducted a LRT where we completely removed each parameter from the model individually. In all cases, the full model, that allowed all parameters to vary, was better than simpler models (Table 2). Code for model selection and simplification is available in Supporting Information 1.

**Table 2:** Result of likelihood ratio tests (LRT) for simplified models including *p*‐values and degrees of freedom (*df*). *p*‐Values < .10 mean more complex model explains significantly more variation than the simplified models. *p*‐Values > .10 mean the simplified model represents the data as well or better than more complex model and should be used

	Carex		Salix	
	*p*	*df*	*p*	*df*
λ held constant	1.01e^−6^	10	0.02	10
λ removed	1.81e^−9^	9	4.76e^−6^	9
α_*ii*_ held constant	2.86e^−6^	10	0.04	10
α_*ii*_ removed	0	9	0	9
α_*ij*_ held constant	0.026	10	0.03	10
α_*ij*_ removed	3.15e^−6^	9	0.04	9

To visualize response surfaces, we used package “plot3D” in R (R Development Core Team 2014). The surface was created using the model to predict the response variable (final cover) for all combinations of the explanatory variables (initial cover of both species). Response surfaces represent modeling predictions, but experimental data are represented in the plots to show model error.

Next, we calculated equilibrium abundances using an analytical solution to the Ricker model (Supporting Information 2). We inserted treatment‐specific parameters and solved for the equilibrium cover of each species for each treatment using the following formulas:

$$\ln N_{c,t}=\;\frac{a_{\mathrm{ss}}\ln\lambda_{c}}{a_{\mathrm{cc}}\;a_{\mathrm{ss}}-\;a_{\mathrm{cs}}\;a_{\mathrm{sc}}\;}$$

$$\ln N_{s,t}=\;\frac{a_{\mathrm{cc}}\ln\lambda_{s}}{a_{\mathrm{ss}}\;a_{\mathrm{cc}}-\;a_{\mathrm{sc}}\;a_{\mathrm{cs}}\;}$$

We simulated our original models over a range of starting cover values to confirm the accuracy of our analytical solution and ensure equilibrium was reached within an ecologically relevant time period, regardless of initial conditions (Figure S2). Equilibrium was reached in all scenarios within 5–10 time steps. We did not consider parameter uncertainty in our model projections.

**Figure 1:**
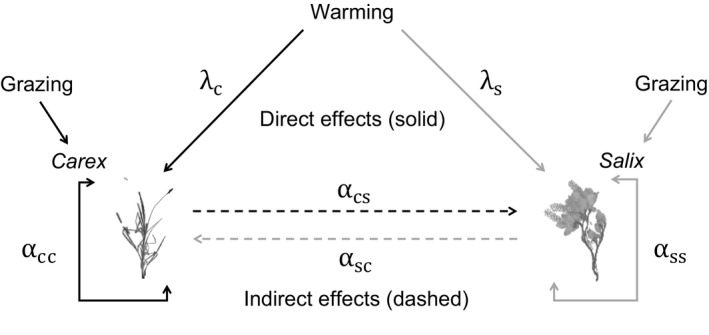
Warming affects each plant directly, by altering the density‐independent growth rate and intraspecific coefficient. Warming can also influence plants indirectly or through alterations to the strength or direction of the interspecific interaction with its neighbor

To address our third objective, we determined the contribution of direct and indirect effects to the overall treatment effect on *Carex* and *Salix* covers. Changes in predicted cover projected by our treatment‐specific models with respect to the current baseline represent the full effect of our experimental warming; it includes both the direct effect of the treatment on each species plus the altered plant–plant interactions that are the indirect effects (Figure 1).

Full effect=Direct effect+Indirect effect

To calculate the direct effect, we returned to our model but held the interspecific parameter constant such that the effect of the neighbor species on the focal species was unchanged by treatment conditions. Parameters for the neighbor species were not allowed to vary by treatment; they were kept at the parameter associated with the ambient, grazed treatment. We used the ambient, grazed treatment as the baseline condition for the model parameters of the competitor species because it represents the scenario that occurs naturally on the landscape. Parameters for the focal species were allowed to vary for the other three treatments as denoted by the subscript (treatment). In the first equation below, *Carex* is treated as the focal species so the parameters for *Salix* (the neighbor) are held constant. In the second equation, *Salix* is treated as the focal species, so the parameters for *Carex* (the neighbor) are held constant.

$$\ln N_{c,t\left[\text{treatment}\right]}=\;\frac{a_{ss\left[\text{ambient,grazed}\right]}\ln\lambda_{c\left[\text{treatment}\right]}}{a_{cc\left[\text{treatment}\right]}\;a_{ss\left[\text{ambient,grazed}\right]}-\;a_{cs\left[\text{treatment}\right]}\;a_{sc\left[\text{ambient,grazed}\right]}\;}$$

$$\ln N_{s,t\left[\text{treatment}\right]}=\;\frac{a_{cc\left[\text{ambient,grazed}\right]}\ln\lambda_{s\left[\text{treatment}\right]}}{a_{ss\left[\text{treatment}\right]}\;a_{cc\left[\text{ambient,grazed}\right]}-\;a_{sc\left[\text{treatment}\right]}\;a_{cs\left[\text{ambient,grazed}\right]}\;}$$

The difference between equilibrium cover for this set of parameters and equilibrium cover projected using baseline parameters is the change in cover resulting from direct effects only, as we removed the possibility of altered indirect effects with a changing climate by holding interspecific effects constant.

## 3 RESULTS

### 3.1 Response surfaces

**Figure 2:**
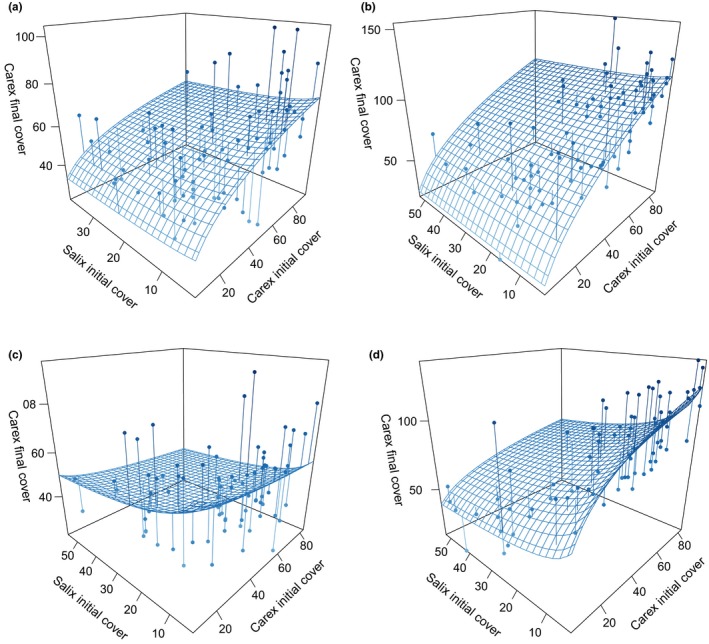
Response surface for modeling predictions of *Carex* cover response to all treatments. (a) Corresponds to ambient, grazed, (b) to ambient, ungrazed, (c) to warmed, grazed, and (d) to warmed, ungrazed. Note that the scale of the vertical axes varies among panels. Also, final cover of *Carex* may exceed 100%. Surface is modeling predictions, points are experimental data, and vertical lines are residuals

In these modeling predictions, *Carex* was largely unaffected by interspecific effects and density of *Salix* under ambient temperatures in both grazed and ungrazed treatments (Figure 2a,b), and under the warmed, grazed treatment (Figure 2c). Only under warmed, ungrazed treatments did *Carex* respond (negatively) to the initial percent cover of *Salix* (Figure 2d).

**Figure 3:**
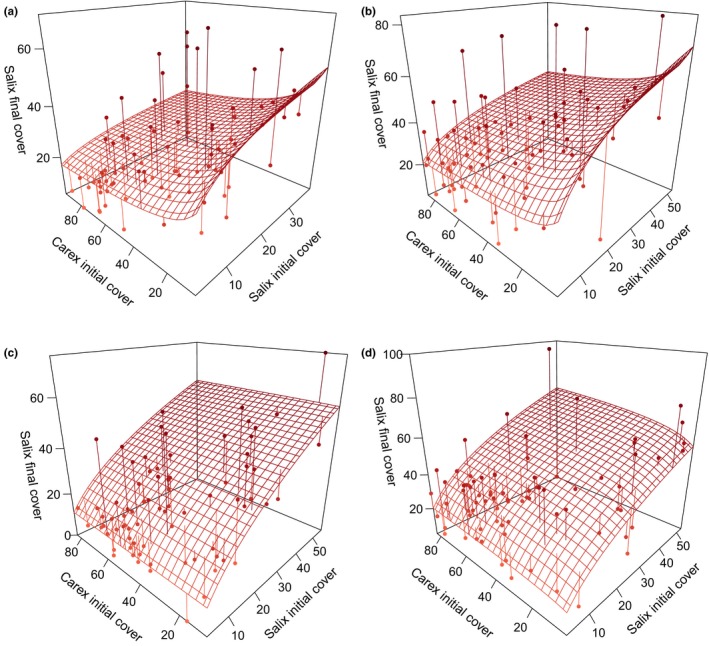
Response surface for modeling predictions of *Salix* cover response to all treatments. (a) Corresponds to ambient, grazed, (b) to ambient, ungrazed, (c) to warmed, grazed, and (d) to warmed, ungrazed. Note that the scale of the vertical axes varies among panels. Surface is modeling predictions, points are experimental data, and vertical lines are residuals

Under ambient conditions in both grazed and ungrazed treatments, *Salix* final percent cover was lower under high *Carex* percent cover due to negative interspecific effects (Figure 3a,b, and Table 3). In the warmed, grazed treatment, *Salix* was unaffected by *Carex* (Figure 3c). In contrast, in the warmed, ungrazed treatment, a slight facilitative effect was apparent in that *Salix* final cover was higher where *Carex* cover was highest (Figure 3d).

**Table 3:** Calculated parameter estimates, 95% confidence limits (CL), residual standard error (RSE), and degrees of freedom (*df*) of best‐fit competition models for each species

Model	Parameter	Estimate	Lower CL	Upper CL	RSE/*df*
*Carex ramenskii*	λ_ambient,grazed_	0.625*	0.449	0.864	0.183/345
	λ_ambient,ungrazed_	1.144*	0.931	1.399	
	λ_warm,grazed_	0.381*	0.263	0.544	
	λ_warm,ungrazed_	0.704*	0.560	0.878	
	α_*cc* ambient,grazed_	0.763*	0.587	0.923	
	α_*cc* ambient,ungrazed_	0.534*	0.413	0.648	
	α_*cc*warm,grazed_	1.087*	0.918	1.247	
	α_*cc* warm,ungrazed_	0.776*	0.672	0.873	
	α_*cs* ambient,grazed_	0.045	−0.058	0.147	
	α_*cs* ambient,ungrazed_	0.023	−0.043	0.087	
	α_*cs* warm,grazed_	0.097	−0.088	0.202	
	α_*cs* warm,ungrazed_	0.162*	0.093	0.232	
*Salix ovalifolia*	λ_ambient,grazed_	0.355*	0.210	0.589	0.151/345
	λ_ambient,ungrazed_	0.534*	0.380	0.750	
	λ_warm,grazed_	0.772*	0.437	1.341	
	λ_warm,ungrazed_	0.934*	0.628	1.378	
	α_*ss* ambient,grazed_	0.791*	0.597	0.972	
	α_*ss* ambient,ungrazed_	0.740*	0.601	0.870	
	α_*ss*warm,grazed_	0.464*	0.248	0.665	
	α_*ss* warm,ungrazed_	0.578*	0.414	0.731	
	α_*sc*ambient,grazed_	0.207*	0.011	0.390	
	α_*sc* ambient,ungrazed_	0.126	−0.001	0.243	
	α_*sc* warm,grazed_	−0.005	−0.248	0.227	
	α_*sc*warm,ungrazed_	−0.089	−0.217	0.034	

*Indicates parameters where confidence intervals do not overlap zero.

Warming affected some model coefficients. Warming decreased the density‐independent growth rate of *Carex* (0.62→0.381), but increased the density‐independent growth rate of *Salix* (0.355→0.772; Table 3). In warmed conditions, *Carex* experienced greater intraspecific competition (0.763→1.087) and *Salix* experienced less intraspecific competition (0.791→0.464; Table 3). *Salix* had a slightly stronger competitive effect on *Carex* when warmed. Notably, *Carex* had a competitive effect on *Salix* in ambient temperatures that shifted to a slight facilitative effect on *Salix* with warming.

Grazing also affected model coefficients. Both *Carex* and *Salix* had higher density‐independent growth rates when ungrazed; however, the increase in growth rate when ungrazed was greater for *Carex* than *Salix*. *Carex* experienced greater intraspecific competition when it was grazed, and *Salix* intraspecific interactions were not affected. There were no consistent directional trends of grazing on interspecific interactions.

### 3.2 Model projections

**Figure 4:**
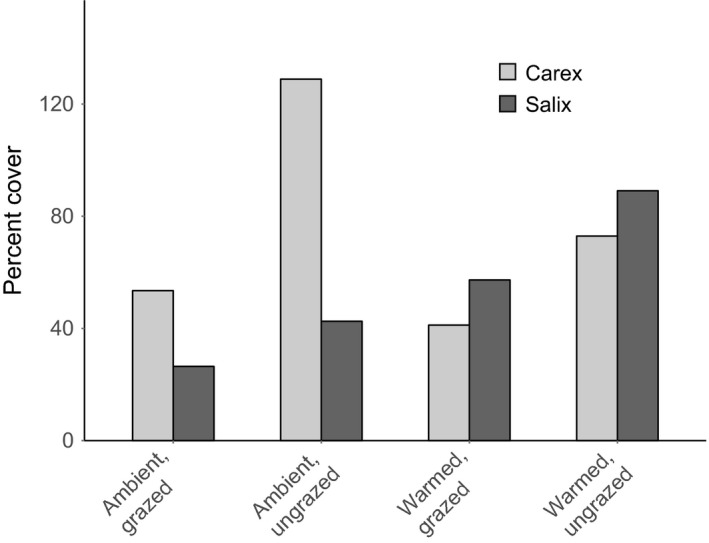
Predicted equilibrium percent cover for each species as calculated using the analytical solution to the model and treatment‐specific parameters

Our analysis of the outcome of species interactions showed coexistence and stable equilibrium (in <10 time steps) in all treatments, though community composition differed across treatments (Figure 4). For the ambient, grazed treatments, our model predicted *Carex* cover would reach equilibrium at 53%, which is similar to the mean cover of *Carex* in control plots (55% ± 16 *SD*). In the ambient, grazed treatment, equilibrium cover of Salix was 26%, which is similar to natural abundances measured in control plots (37% ± 12 *SD*).

In both grazed and ungrazed ambient treatments, *Carex* was the dominant species. In the ambient, ungrazed treatment, equilibrium for *Carex* was 129% and 43% for *Salix*. However, in warmed treatments, *Salix* abundance increased and became the dominant species. In the warmed, grazed treatment, *Carex* equilibrium percent cover was 41% while *Salix* reached 57%. In the warmed, ungrazed treatment, *Carex* equilibrium percent cover was 73% and *Salix* equilibrium percent cover was 89%.

### 3.3 Direct and indirect effects

**Figure 5:**
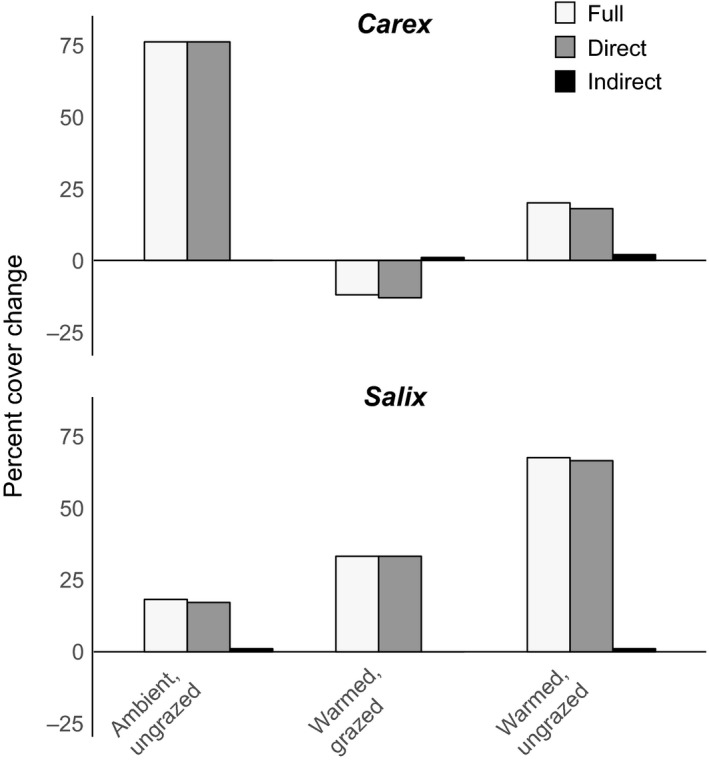
Contribution of full effect, direct, and indirect effects to change in cover between the natural condition (ambient, grazed) and the other three temperature, grazing combination treatments. Negative values indicate a decrease in cover from the natural condition. The full effect is the sum of direct and indirect bars

We found that the direct effects of warming and grazing on plant growth were greater than indirect effects in all treatments for both species (Figure 5). Direct effects accounted for 90%–100% of the total predicted changes in equilibrium cover between the ambient, grazed condition and the other conditions. Indirect effects accounted for only 0%–10% of the predicted changes in cover.

## 4 DISCUSSION

Our study sought to determine how the abundances of *Carex* and *Salix* might change under warmed conditions, with and without herbivory. Similar to other studies investigating the interaction between sedges and woody plants (Elliott & Henry, 2011), we found that *Carex* was the dominant species under ambient conditions based on modeling predictions as well as raw experimental data (Figure S3). Using experimental data to parameterize competition models, we found that models predicted that *Salix* will overtake *Carex* as the dominant species in the system under warmed conditions whether the system is grazed or ungrazed. While this result is surprising because *Salix* currently occupies a smaller percent of the landscape, it is consistent with other studies from across the Arctic showing that warming causes a community shift (i.e., a decline in sedges and increase in deciduous shrubs; Chapin, Shaver, Giblin, Nadelhoffer, & Laundre, 1995; Sturm et al., 2001). We found that direct effects of climate change on individual plant species vital rates were substantially more important than the indirect effects, mediated through species interactions.

### 4.1 Effect of warming

Species with different functional types or growth strategies may respond differently to warmed conditions (Burt, Dunn, Nichols, & Sanders, 2014; Post, 2013; Post & Pedersen, 2008). We found that warming increased the growth rate of *Salix* but decreased the growth rate of *Carex*. Our models are consistent with previous studies that have found that warming increases the abundance of *Salix* and other deciduous shrubs across the Arctic (Elmendorf et al., 2012; Myers‐Smith et al., 2011; Sturm et al., 2001; Tape et al., 2006), while warming decreases the abundance of *Carex* and other graminoids (Chapin, Bret‐Harte, Hobbie, & Zhong, 1996; Chapin & Shaver, 1985; Chapin et al., 1995). Although our study did not identify a physiological mechanism for this shift, results from our models suggest that an increase in deciduous shrubs and decrease in graminoids across the Arctic may be a direct response of these species to warming rather than an indirect response, such as competitive release of *Salix* or suppression of *Carex*.

### 4.2 Effect of herbivory

In other systems, grazing can mitigate climate change effects by preventing a change in community composition or limiting shrub expansion (Kaarlejärvi et al., 2015; Olofsson et al., 2009; Post & Pedersen, 2008). In our study, herbivory did not prevent a community shift, but it substantially reduced the cover of both species. Herbivory in our system may not prevent a shift in species dominance with warming for two reasons. First, other studies showing that herbivores can reduce shrub growth with warming have focused on mammalian herbivores that prefer to browse shrubs (Bråthen & Oksanen, 2001; Eskelinen & Oksanen, 2006; Post & Pedersen, 2008). In our experiment, we simulated grazing on plants in proportion to their cover; however, geese would likely primarily graze sedges, which further support the finding that geese would not mediate a woody plant increase in our system. Second, the amount of vegetation removed by geese may not be as substantial as that removed by the mammalian herbivores (Kaarlejärvi et al., 2015).

### 4.3 Direct and indirect effects

In modeling projections of our system, direct effects of warming were more important than the indirect effects of warming mediated by plant–plant interactions, as has been found in other graminoid‐dominated systems (Chu et al., 2016). Direct effects explained an average of 97% of the total change in cover. These direct effects, which resulted in *Salix* becoming the dominant species, were driven by a reduction in the density‐independent growth rate of *Carex* and an increase in the density‐independent growth rate of *Salix* in warmed conditions. They were also driven by a reduction in intraspecific competition for *Salix*, but an increase in intraspecific competition for *Carex*.

More specifically, warming and grazing together had negative effects on *Carex* by reducing the growth rate and increasing the per capita effect of intraspecific competition. Therefore, *Carex* increased substantially in the ambient, ungrazed condition and declined in the warmed, grazed. In contrast, warming positively affected *Salix*, and grazing did not affect it as strongly as it affected *Carex*. Because warming increased the growth rate and reduced intraspecific competition, our models project that *Salix* will eventually become the dominant species in warmed conditions.

Indirect effects only explained 8%–10% of the total change in *Carex* cover and 0%–2% of *Salix*. Weak interspecific interactions are common in stressful alpine and Arctic systems (Callaway et al., 2002; Cavieres et al., 2014). Consistent with this finding, most of our estimated interspecific coefficients overlapped zero. Holding these interspecific interactions constant in the model and considering only direct effects had negligible effects on projected outcomes.

### 4.4 Limitations

Our model of plant responses to warming reflects changes we observed in treatments that increased temperatures by 1.75°C during the spring and summer only. While our results show a shift in plant dominance even under this minimal increase, our predictions are limited to this small, consistent increase. Continually increasing temperatures may further alter the community beyond our predictions. Furthermore, climate change could have other effects on this system, such as increased soil salinity or sedimentation rates due to more extreme flood events and sea level rise, that we do not address and could be contrary to the effects of warming alone (Person & Ruess, 2003; Terenzi et al., 2014).

There were some limitations to our herbivory treatments. The dominant herbivores in our system are geese. While some goose species, such as black brant, forage almost exclusively on *Carex* during the breeding season, other species, like greater white‐fronted geese and cackling geese, are less restrictive in their diets and their populations are increasing (Fischer & Stehn, 2014). We simulated *Salix* herbivory based on *Carex* black brant herbivory because no studies have yet quantified how much *Salix* geese or mammalian herbivores at the site consume. However, because warming increased *Salix* cover above *Carex* with or without herbivory, this treatment did not qualitatively change our conclusions. Finally, we only simulated tissue removal and fecal addition. Future studies should investigate the importance of goose trampling on these species.

### 4.5 Future climate and herbivore change

Our models suggest that an instantaneous increase in temperature of 1.75°C could result in a shift from sedge to deciduous shrub dominance in an important brood‐rearing habitat for migratory geese in 5–10 time steps regardless of initial conditions, suggesting we might observe this change over decadal timescales. *C. ramenskii* is an important goose forage species in this coastal wetland ecosystem, and a shift toward a dwarf shrub‐dominated landscape would reduce the availability of high‐quality forage for the migratory geese that utilize this habitat (Sedinger & Raveling, 1984). The amount of high‐quality forage consumed is a strong predictor of gosling survival, thus a shift toward less‐nutritious forage, such as *Salix*, could further reduce this already declining black brant population (Sedinger & Chelgren, 2007; Sedinger et al., 2016). With climate change, late arrival to the breeding grounds by geese (in comparison with date of green‐up), migration to more suitable environments, or continued population decline could result in reduced herbivore pressure and potentially further increase *Salix* cover and reduce the availability of preferred forage species (Sedinger et al., 2016; Ward et al., 2005, 2016). Managers may wish to consider the impacts of shrub expansion on the active floodplain when assessing habitat availability for goose herbivores. Finally, the consequence of novel herbivores moving into the system, such as moose (Tape et al., 2016) or snow geese, is unknown and should be considered.

### 4.6 Conclusions

Climate change can affect species vital rates and interactions, and the effects of herbivory may be important in mediating climate change effects on plant communities. The results of our study suggest that an increase in less than 2°C could cause a shift in dominance from sedges to dwarf shrubs on the coast of western Alaska. This shift will likely be a result of the direct effects of warming and not a result of changes to plant–plant interactions or competitive release. Our results provide evidence for an increase in woody plant abundance on the subarctic coast and add to literature suggesting that direct effects of warming are stronger in systems where species have different growth strategies. If direct effects are more important than indirect effects in other Arctic systems, this improves our understanding of how woody plant abundance is increasing. Further, we show that goose herbivores may not be able to mitigate shrubification in a manner similar to mammalian herbivores. Future changes in the relative abundance of these plant species have implications for how many herbivores and what types of herbivores these landscapes can support.

## CONFLICT OF INTEREST

None declared.

## AUTHOR CONTRIBUTIONS

LC, KB, and PA conceived and designed the experiments. LC performed the experiments. LC and PA analyzed the data. LC, KB, and PA wrote and edited the paper.

## DATA ACCESSIBILITY

Data are available through the Arctic Data Center at https://doi.org/10.18739/a2mp11.
