# Supplementary material for: Direct effects of warming increase woody plant abundance in a subarctic wetland
Source: Ecol Evol. 2018 Feb 11;8(5):2868–79. doi: 10.1002/ece3.3902 (PMC5838087; doi:10.1002/ece3.3902)
Supplement: Supplementary file 1 [file ECE3-8-2868-s001.docx]

**Supporting Information**


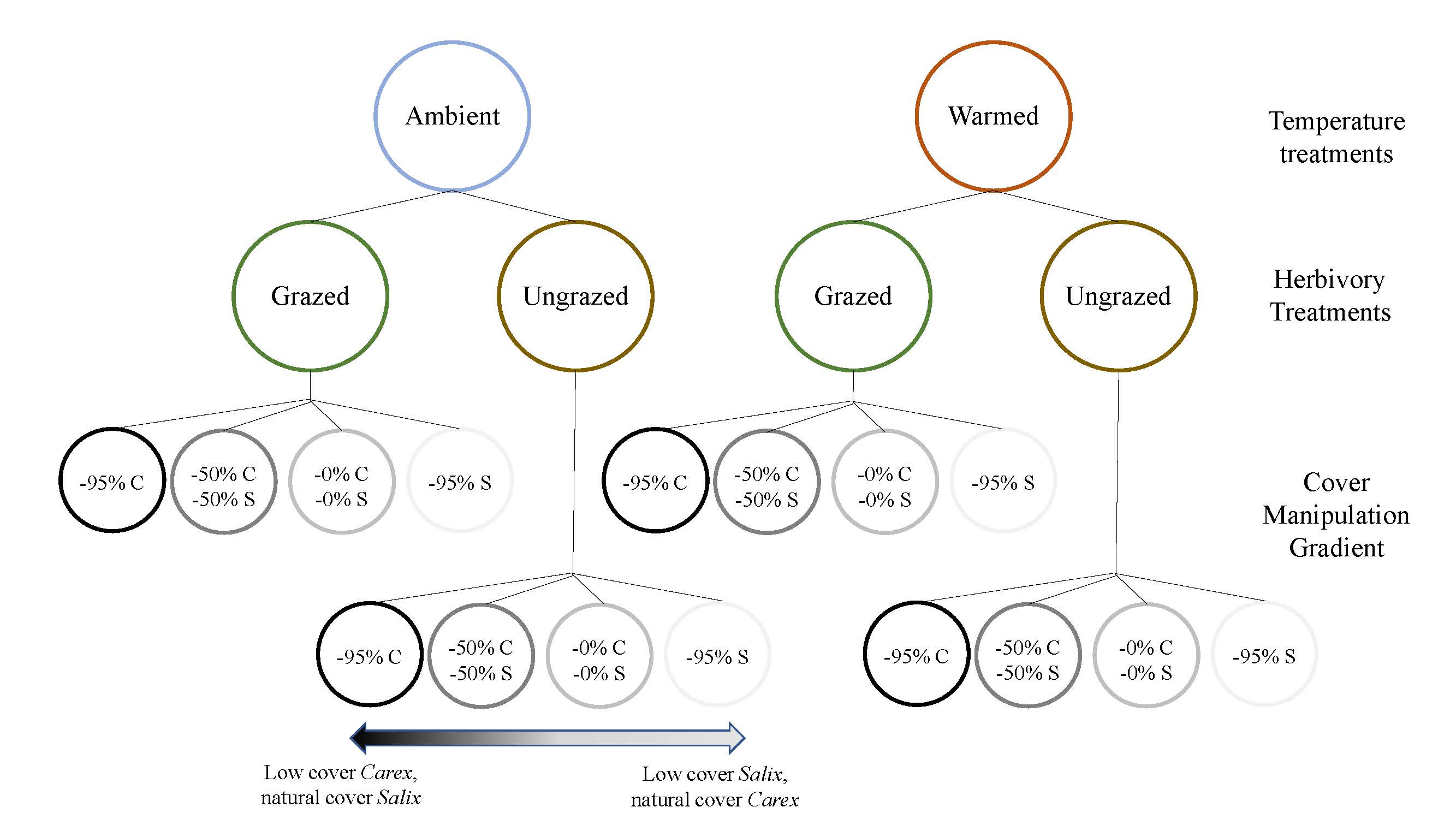


**Figure S1**. Our experimental design contained of 5 replicates of 4 treatments created from the factorial combination of (+/-) warming and (+/-) grazing. Nested within each treatment were four possible cover manipulations to create a cover gradient for both species.

**Table S1**. Relevant models from the literature suggested by Inouye 2001 and reparametrized by Hart & Marshall 2013 to include an explicit term for intraspecific competition (rather than a carrying capacity). The response variable for all models N*_i,t+1_* when N*_i,t+1_ =* N*_i,t_ f(X_t_ ,Y_t_)*, λ is the growth rate, α*_ii_* and α*_ij_* are intra and interspecific competition coefficients respectively, N*_i,t_* and N*_j,t_* are initial percent cover of the focal species and the competitor respectively, and b is a parameter that allows for a more flexible fit. Only models one, three, and four could be fit for both species. The rest failed to converge.

| f(X_t_,Y_t_) |
| --- |
| $\boldsymbol{\lambda}\boldsymbol{e}^{\boldsymbol{-\alpha}_{\boldsymbol{ii}}\boldsymbol{N}_{\boldsymbol{i,t}}\boldsymbol{-}\boldsymbol{\alpha}_{\boldsymbol{ij}} \boldsymbol{N}_{\boldsymbol{j,t}}}$ * |
| $\boldsymbol{\lambda/(1+}\boldsymbol{\alpha}_{\boldsymbol{ii}}\boldsymbol{N}_{\boldsymbol{i,t}}\boldsymbol{+}\boldsymbol{\alpha}_{\boldsymbol{ij}}\boldsymbol{N}_{\boldsymbol{j,t}}\boldsymbol{)}$ |
| $\boldsymbol{\lambda}\boldsymbol{e}^{\boldsymbol{-\alpha}_{\boldsymbol{ii}}\mathbf{ln}\boldsymbol{(}\boldsymbol{N}_{\boldsymbol{i,t}}\boldsymbol{)-}\boldsymbol{\alpha}_{\boldsymbol{ij}}\mathbf{ln}\boldsymbol{(}\boldsymbol{N}_{\boldsymbol{j,t}}\boldsymbol{)}}$ * |
| $\boldsymbol{1+\lambda(1-}\boldsymbol{\alpha}_{\boldsymbol{ii}}\boldsymbol{N}_{\boldsymbol{i,t}}\boldsymbol{-}\boldsymbol{\alpha}_{\boldsymbol{ij}}\boldsymbol{N}_{\boldsymbol{j,t}}\boldsymbol{)}$ * |
| $\boldsymbol{\lambda/(1+}{\boldsymbol{N}_{\boldsymbol{i,t}}}^{\boldsymbol{\alpha}_{\boldsymbol{ii}}}\boldsymbol{+}{\boldsymbol{\alpha}_{\boldsymbol{ij}}}^{\boldsymbol{N}_{\boldsymbol{j,t}}}\boldsymbol{)}$ |
| $\boldsymbol{\lambda/}{\boldsymbol{1+(}\boldsymbol{\alpha}_{\boldsymbol{ii}}\boldsymbol{N}_{\boldsymbol{i,t}}\boldsymbol{+}\boldsymbol{\alpha}_{\boldsymbol{ij}}\boldsymbol{N}_{\boldsymbol{j,t}}\boldsymbol{)}}^{\boldsymbol{b}}$ |
| $\boldsymbol{\lambda/}{\boldsymbol{(1+}\boldsymbol{\alpha}_{\boldsymbol{ii}}\boldsymbol{N}_{\boldsymbol{i,t}}\boldsymbol{+}\boldsymbol{\alpha}_{\boldsymbol{ij}}\boldsymbol{N}_{\boldsymbol{j,t}}\boldsymbol{)}}^{\boldsymbol{b}}$ |

**
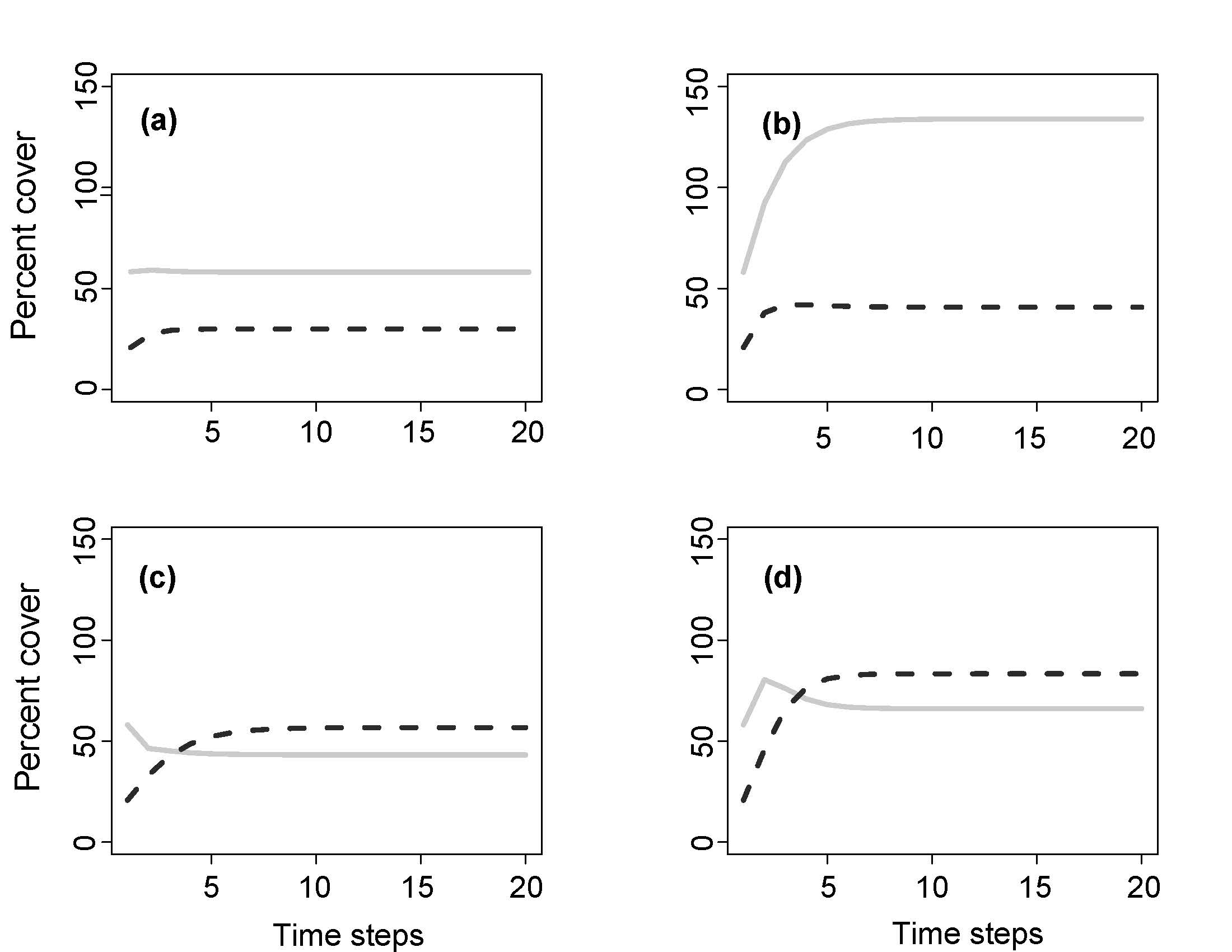
**

**Figure S2**. We used models to predict the equilibrium cover of *Carex* (solid, light gray) and *Salix* (dashed, dark gray) for each of our treatment combinations: (a) ambient, grazed, (b) ambient, ungrazed, (c) warmed, grazed, and (d) warmed, ungrazed. We tested the equilibrium outcomes of our models under a range of initial cover combinations: 1) 5% *Carex*, 95% *Salix* 2) 95% *Carex*, 5% *Salix*, 2) 50% *Carex*, 50% *Salix*, 4) and natural abundance 55% *Carex*, 37% *Salix*. Here, we only show the natural abundance as the initial condition because the same equilibrium was reached after 5-10 time steps, regardless of initial values.

**
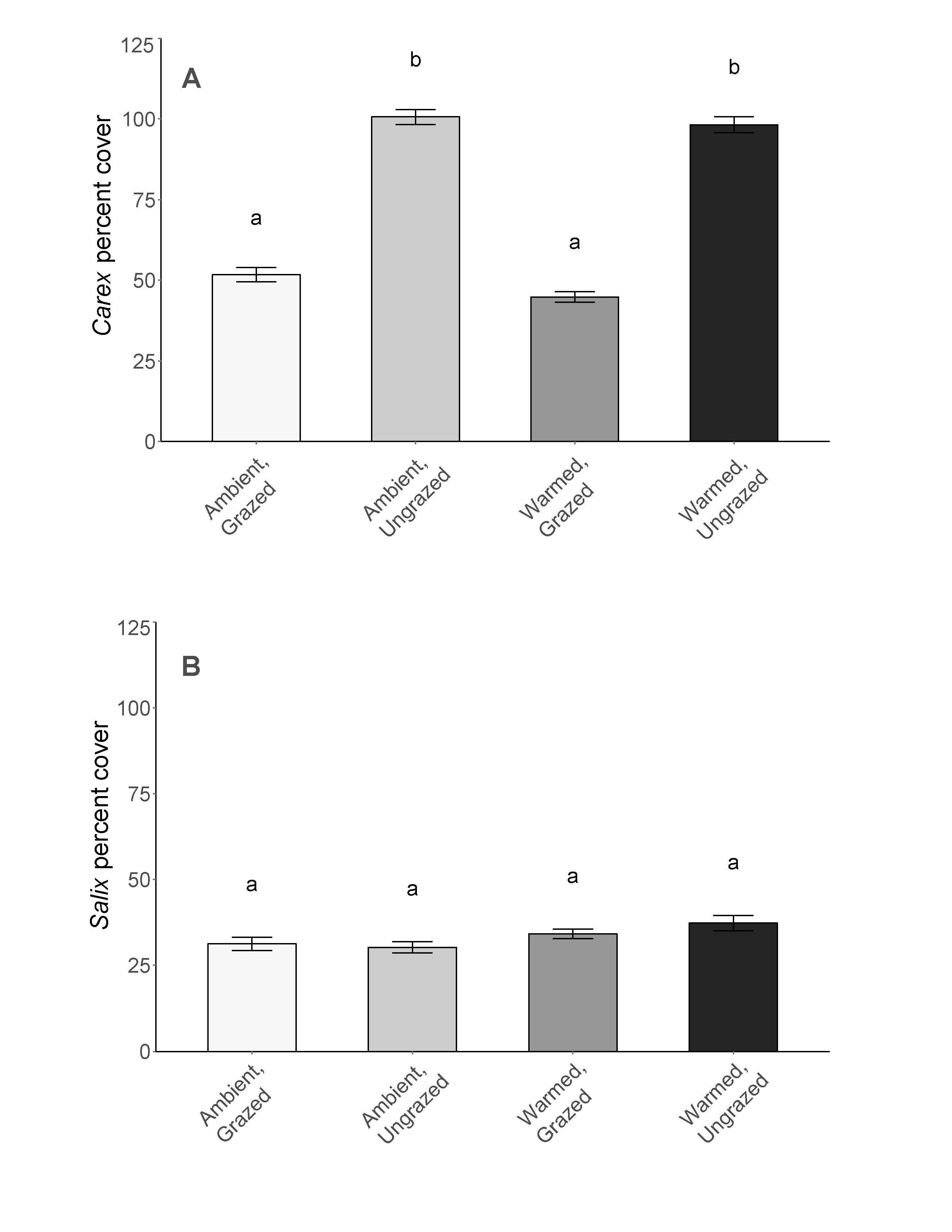
**

**Figure S3**. Experimental percent cover of *Carex* (A) and *Salix* (B) in each of the four treatments at the end the second growing season. The cover of *Carex* is significantly greater in ungrazed plots than in grazed plots, but it is slightly lower in warmed conditions. The cover of *Salix* is not significantly different among treatments, though is slightly greater under warmed conditions.

**Supporting Information 1. Reproducible R code for analysis**

#find and cite dataset at:

#Gray Carlson, L. (2017). Point-count cover of Carex ramenskii and Salix ovalifolia under #warmed and grazed conditions. Yukon-Kuskokwim Delta, Alaska, 2015-2016 [Data set]. #Arctic Data Center. https://doi.org/10.18739/a2mp11

path.coverdata<-("C:\\Users\\grad\\Desktop") #please reset working directory for your data source

setwd(path.coverdata)

cover<-read.csv("arctic-data.14103.1.csv",header=T) #import neighborhood cover dataset from above citation

head(cover)

str(cover)

cover.frame<-data.frame(cover) #convert to a dataframe

c.cov<-cover.frame[c("plot_num","warm_treatment","herb_treatment","Nct0","Nst0","Nct1")] #create data frames with only data needed for each model

s.cov<-cover.frame[c("plot_num","warm_treatment","herb_treatment","Nct0","Nst0","Nst1")]

Nct1<-c.cov$Nct1

Nct0<-c.cov$Nct0

Nst0<-c.cov$Nst0

c.cov$env.fac = as.factor(paste(c.cov$warm_treatment,c.cov$herb_treatment,sep = ".")) #add column containing factorial combination of warming + herbivory

Nst1<-s.cov$Nst1

s.cov$senv.fac = as.factor(paste(s.cov$warm_treatment,s.cov$herb_treatment,sep = ".")) #add column containing factorial combination of warming + herbivory

#test models#

###################Model 2#########################

###Carex Ricker 2###

carex.model2 = nls(formula=Nct1 ~ Nct0*(lambda[env.fac] * exp(-alpha.cc[env.fac]*Nct0 - alpha.cs[env.fac]*Nst0)),

data=c.cov,

start = list(lambda = c(5,5,5,5), alpha.cc = c(1,1,1,1), alpha.cs = c(0,0,0,0)),

algorithm = "port")

summary(carex.model2)

#structure is formula calling data from specified data frame

#[env.fac] term allows this param to vary by treatment ie amb/graz, amb/ungraz, warm/graz, warm/ungraz

#start is a list of starting "guesses" for each parameter, modifying these (within reason) does not alter parameter estimate (ie model will not converge if starting value is 10000000 but WILL be the same if you guess 10)

#algorithm, changing algorithm did not alter params or change covergence status

###Salix Ricker 2###

salix.model2 = nls(formula=Nst1 ~ Nst0*(lambda[senv.fac] * exp(-alpha.ss[senv.fac]*Nst0 - alpha.sc[senv.fac]*Nct0)),

data=s.cov,

start = list(lambda = c(5,5,5,5), alpha.ss = c(1,1,1,1), alpha.sc = c(0,0,0,0)),

algorithm = "port")

summary(salix.model2)

nls.control(maxiter = 1000) #increasing iterations to max did not allow convergence

###################Model 3#########################

###Carex Ricker 3###

carex.model3 = nls(formula=Nct1 ~ Nct0*(lambda[env.fac]/(1 + alpha.cc[env.fac]*Nct0 + alpha.cs[env.fac]*Nst0)),

data=c.cov,

start = list(lambda = c(11,6,9,4), alpha.cc = c(9,7,5,7), alpha.cs = c(1,0,0,0)),

algorithm = "port")

summary(carex.model3) #convergence failure#

#changing starting value did not allow convergence

###Salix Ricker 3###

salix.model3 = nls(formula=Nst1 ~ Nst0*(lambda[senv.fac]/(1 + alpha.ss[senv.fac]*Nst0 + alpha.sc[senv.fac]*Nct0)),

data=s.cov,

start = list(lambda = c(5,5,5,5), alpha.ss = c(1,1,1,1), alpha.sc = c(0,0,0,0)),

algorithm = "port")

summary(salix.model3) #convergence failure#

###################Model 4#########################

###Carex Ricker 4###

carex.model4 = nls(formula=Nct1 ~ Nct0*(lambda[env.fac]*exp((-alpha.cc[env.fac]*log(Nct0))-(alpha.cs[env.fac]*log(Nst0)))),

data=c.cov,

start = list(lambda = c(5,5,5,5), alpha.cc = c(1,1,1,1), alpha.cs = c(0,0,0,0)),

algorithm = "port")

summary(carex.model4)

###Salix Ricker 4###

salix.model4 = nls(formula=Nst1 ~ Nst0*(lambda[senv.fac]*exp((-alpha.ss[senv.fac]*log(Nst0))-(alpha.sc[senv.fac]*log(Nct0)))),

data=s.cov,

start = list(lambda = c(5,5,5,5), alpha.ss = c(1,1,1,1), alpha.sc = c(0,0,0,0)),

algorithm = "port")

summary(salix.model4)

###################Model 5#########################

###Carex Ricker 5###

carex.model5 = nls(formula=Nct1 ~ Nct0*(1 + lambda[env.fac]*(1-alpha.cc[env.fac]*Nct0-alpha.cs[env.fac]*Nst0)),

data=c.cov,

start = list(lambda = c(5,5,5,5), alpha.cc = c(1,1,1,1), alpha.cs = c(0,0,0,0)),

algorithm = "port")

summary(carex.model5)

###Salix Ricker 5###

salix.model5 = nls(formula=Nst1 ~ Nst0*(1 + lambda[senv.fac]*(1-(alpha.ss[senv.fac]*Nst0)-(alpha.sc[senv.fac]*Nct0))),

data=s.cov,

start = list(lambda = c(5,5,5,5), alpha.ss = c(1,1,1,1), alpha.sc = c(0,0,0,0)),

algorithm = "port")

summary(salix.model5)

###################Model 6#########################

###Carex Ricker 6###

carex.model6 = nls(formula=Nct1 ~ Nct0*(lambda[env.fac]/(1 + Nct0^(alpha.cc[env.fac])+Nst0^(alpha.cs[env.fac]))),

data=c.cov,

start = list(lambda = c(11,6,9,4), alpha.cc = c(9,7,5,7), alpha.cs = c(1,0,0,0)),

algorithm = "default",

control = list(maxiter = 1000))

summary(carex.model6) #convergence failure#

salix.model6 = nls(formula=Nst1 ~ Nst0*(lambda[senv.fac]/(1 + Nst0^(alpha.ss[senv.fac])+Nct0^(alpha.sc[senv.fac]))),

data=s.cov,

start = list(lambda = c(11,6,9,4), alpha.ss = c(9,7,5,7), alpha.sc = c(1,0,0,0)),

algorithm = "port",

control = list(maxiter = 1000))

summary(salix.model6) #convergence failure#

#increased iterations or changing start valued did not allow convergence

###################Model 7#########################

###Carex Ricker 7###

carex.model7 = nls(formula=Nct1 ~ Nct0*(lambda[env.fac]/1 + (((alpha.cc[env.fac]*Nct0)+(alpha.cs[env.fac]*Nst0))^b)),

data=c.cov,

start = list(lambda = c(5,5,2,3), alpha.cc = c(3,3,2,3), alpha.cs = c(0,0,0,0), b = 2),

algorithm = "default")

summary(carex.model7)

##failed##

###Salix Ricker 7###

salix.model7 = nls(formula=Nst1 ~ Nst0*(lambda[senv.fac]/1 + (((alpha.ss[senv.fac]*Nst0)+(alpha.sc[senv.fac]*Nct0))^b)),

data=s.cov,

start = list(lambda = c(5,5,2,3), alpha.ss = c(3,3,2,3), alpha.sc = c(0,0,0,0), b = 2),

algorithm = "default")

summary(salix.model7)

##failed##

#increased iterations or changing start valued did not allow convergence, nor did changing algorithm

###################Model 8#########################

###Carex Ricker 8###

carex.model8 = nls(formula=Nct1 ~ Nct0*(lambda[env.fac]/(1 + (alpha.cc[env.fac]*Nct0)+(alpha.cs[env.fac]*Nst0))^b[env.fac]),

data=c.cov,

start = list(lambda = c(5,5,2,3), alpha.cc = c(3,3,2,3), alpha.cs = c(0,0,0,0), b = c(2,2,2,2)),

algorithm = "default")

summary(carex.model8)

##failed##

###Salix Ricker 8###

salix.model8 = nls(formula=Nst1 ~ Nst0*(lambda[senv.fac]/(1 + (alpha.ss[senv.fac]*Nst0)+(alpha.sc[senv.fac]*Nct0)^b[senv.fac])),

data=s.cov,

start = list(lambda = c(5,5,2,3), alpha.ss = c(3,3,2,3), alpha.sc = c(0,0,0,0), b = c(2,2,2,2)),

algorithm = "default")

summary(salix.model8)

##failed##

#increased iterations or changing start valued did not allow convergence, nor did changing algorithm

#install.packages("AICcmodavg") #install this package if you do not already have it, note this only works in version 3.3.3 or later

library(AICcmodavg)

###create candidate model list for getting AICc table###

Cand.c.mod <- list()

Cand.c.mod[[1]] <- carex.model2

Cand.c.mod[[2]] <- carex.model4

Cand.c.mod[[3]] <- carex.model5

###create vector of the model names

cmod.name <- c("carex.model2","carex.model4","carex.model5")

###get AICc table for ranking predictive models--------------------------

c.aictab<-aictab(cand.set = Cand.c.mod, modnames = cmod.name, second.ord = TRUE, sort = TRUE)

print(c.aictab)

#write.csv(c.aictab,file = "carexmodelaic.csv",row.names = FALSE,col.names = TRUE)

###create candidate model list for getting AICc table###

Cand.s.mod <- list()

Cand.s.mod[[1]] <- salix.model2

Cand.s.mod[[2]] <- salix.model4

Cand.s.mod[[3]] <- salix.model5

###create vector of the model names

smod.name <- c("salix.model2","salix.model4","salix.model5")

###get AICc table for ranking predictive models--------------------------

s.aictab<-aictab(cand.set = Cand.s.mod, modnames = smod.name, second.ord = TRUE, sort = TRUE)

print(s.aictab)

#write.csv(s.aictab,file = "salixmodelaic.csv",row.names = FALSE,col.names = TRUE)

############################

#for both C and S, model4 is the best fitting by AICc#

############################

##########LRT#################

####likelihood ratio tests of reduced models########

#Carex LRT#

###################Model 4#########################

###Carex Ricker 4###

carex.model4 = nls(formula=Nct1 ~ Nct0*(lambda[env.fac]*exp((-alpha.cc[env.fac]*log(Nct0))-(alpha.cs[env.fac]*log(Nst0)))),

data=c.cov,

start = list(lambda = c(5,5,5,5), alpha.cc = c(1,1,1,1), alpha.cs = c(0,0,0,0)),

algorithm = "port")

summary(carex.model4)

# model which does not allow lambda to vary by treatment

carex.model4r1.1 = nls(formula=Nct1 ~ Nct0*(lambda*exp((-alpha.cc[env.fac]*log(Nct0))-(alpha.cs[env.fac]*log(Nst0)))),

data=c.cov,

start = list(lambda = 1, alpha.cc = c(1,1,1,1), alpha.cs = c(0,0,0,0)),

algorithm = "port")

summary(carex.model4r1.1)

######tested model not varying lambda######

anova(carex.model4,carex.model4r1.1)

Qcr1 = -2 * (logLik(carex.model4r1.1) - logLik(carex.model4))

df.Qcr1 = df.residual(carex.model4r1.1) - df.residual(carex.model4)

1 - pchisq(Qcr1, df.Qcr1)

# model which does not allow alpha.cc to vary by tx

carex.model4r2.2 = nls(formula=Nct1 ~ Nct0*(lambda[env.fac]*exp((-alpha.cc*log(Nct0))-(alpha.cs[env.fac]*log(Nst0)))),

data=c.cov,

start = list(lambda = c(5,5,5,5), alpha.cc = 1, alpha.cs = c(0,0,0,0)),

algorithm = "port")

summary(carex.model4r2.2)

######tested model not varying alpha.cc######

anova(carex.model4,carex.model4r2.2)

Qcr2 = -2 * (logLik(carex.model4r2.2) - logLik(carex.model4))

df.Qcr2 = df.residual(carex.model4r2.2) - df.residual(carex.model4)

1 - pchisq(Qcr2, df.Qcr2)

# model which does not allow alpha.cs to vary by ttreatment

carex.model4r3.3 = nls(formula=Nct1 ~ Nct0*(lambda[env.fac]*exp((-alpha.cc[env.fac]*log(Nct0))-(alpha.cs*log(Nst0)))),

data=c.cov,

start = list(lambda = c(5,5,5,5), alpha.cc = c(1,1,1,1), alpha.cs = 0),

algorithm = "port")

summary(carex.model4r3.3)

######tested model not varying alpha.cs######

anova(carex.model4,carex.model4r3.3)

Qcr3 = -2 * (logLik(carex.model4r3.3) - logLik(carex.model4))

df.Qcr3 = df.residual(carex.model4r3.3) - df.residual(carex.model4)

1 - pchisq(Qcr3, df.Qcr3)

#model which removed lambda

carex.model4r1 = nls(formula=Nct1 ~ Nct0*(exp((-alpha.cc[env.fac]*log(Nct0))-(alpha.cs[env.fac]*log(Nst0)))),

data=c.cov,

start = list(alpha.cc = c(1,1,1,1), alpha.cs = c(0,0,0,0)),

algorithm = "port")

summary(carex.model4r1)

######tested model removing lambda######

anova(carex.model4,carex.model4r1)

Qcr1 = -2 * (logLik(carex.model4r1) - logLik(carex.model4))

df.Qcr1 = df.residual(carex.model4r1) - df.residual(carex.model4)

1 - pchisq(Qcr1, df.Qcr1)

#model which removed alpha.cc

carex.model4r2 = nls(formula=Nct1 ~ Nct0*(lambda[env.fac]*exp((-(alpha.cs[env.fac]*log(Nst0))))),

data=c.cov,

start = list(lambda = c(5,5,5,5), alpha.cs = c(0,0,0,0)),

algorithm = "port")

summary(carex.model4r2)

######tested model removing alpha.cc######

anova(carex.model4,carex.model4r2)

Qcr2 = -2 * (logLik(carex.model4r2) - logLik(carex.model4))

df.Qcr2 = df.residual(carex.model4r2) - df.residual(carex.model4)

1 - pchisq(Qcr2, df.Qcr2)

#model which removed alpha.cs

carex.model4r3 = nls(formula=Nct1 ~ Nct0*(lambda[env.fac]*exp((-alpha.cc[env.fac]*log(Nct0)))),

data=c.cov,

start = list(lambda = c(5,5,5,5), alpha.cc = c(1,1,1,1)),

algorithm = "port")

summary(carex.model4r3)

######tested model removing alpha.cs######

anova(carex.model4,carex.model4r3)

Qcr3 = -2 * (logLik(carex.model4r3) - logLik(carex.model4))

df.Qcr3 = df.residual(carex.model4r3) - df.residual(carex.model4)

1 - pchisq(Qcr3, df.Qcr3)

#Salix LRT#

###Salix Ricker 4###

salix.model4 = nls(formula=Nst1 ~ Nst0*(lambda[senv.fac]*exp((-alpha.ss[senv.fac]*log(Nst0))-(alpha.sc[senv.fac]*log(Nct0)))),

data=s.cov,

start = list(lambda = c(5,5,5,5), alpha.ss = c(1,1,1,1), alpha.sc = c(0,0,0,0)),

algorithm = "port")

summary(salix.model4)

#model which did not allow lambda to vary by treatment

salix.model4r1.1 = nls(formula=Nst1 ~ Nst0*(lambda*exp((-alpha.ss[senv.fac]*log(Nst0))-(alpha.sc[senv.fac]*log(Nct0)))),

data=s.cov,

start = list(lambda = 1, alpha.ss = c(1,1,1,1), alpha.sc = c(0,0,0,0)),

algorithm = "port")

summary(salix.model4r1.1)

#tested model not varying lambda

anova(salix.model4,salix.model4r1.1)

Qsr1 = -2 * (logLik(salix.model4r1.1) - logLik(salix.model4))

df.Qsr1 = df.residual(salix.model4r1.1) - df.residual(salix.model4)

1 - pchisq(Qsr1, df.Qsr1)

#model which did not allow alpha.ss to vary by treatment

salix.model4r2.2 = nls(formula=Nst1 ~ Nst0*(lambda[senv.fac]*exp((-alpha.ss*log(Nst0))-(alpha.sc[senv.fac]*log(Nct0)))),

data=s.cov,

start = list(lambda = c(5,5,5,5), alpha.ss = 1, alpha.sc = c(0,0,0,0)),

algorithm = "port")

summary(salix.model4r2.2)

######tested model not varying alpha.ss######

anova(salix.model4,salix.model4r2.2)

Qsr2 = -2 * (logLik(salix.model4r2.2) - logLik(salix.model4))

df.Qsr2 = df.residual(salix.model4r2.2) - df.residual(salix.model4)

1 - pchisq(Qsr2, df.Qsr2)

#model which did not allow alpha.sc to vary by treatment

salix.model4r3.3 = nls(formula=Nst1 ~ Nst0*(lambda[senv.fac]*exp((-alpha.ss[senv.fac]*log(Nst0))-(alpha.sc*log(Nct0)))),

data=s.cov,

start = list(lambda = c(5,5,5,5), alpha.ss = c(1,1,1,1), alpha.sc = 0),

algorithm = "port")

summary(salix.model4r3.3)

######tested model not varying alpha.sc######

anova(salix.model4,salix.model4r3.3)

Qsr3 = -2 * (logLik(salix.model4r3.3) - logLik(salix.model4))

df.Qsr3 = df.residual(salix.model4r3.3) - df.residual(salix.model4)

1 - pchisq(Qsr3, df.Qsr3)

######model reduced by removing lambda######

salix.model4r1 = nls(formula=Nst1 ~ Nst0*(exp((-alpha.ss[senv.fac]*log(Nst0))-(alpha.sc[senv.fac]*log(Nct0)))),

data=s.cov,

start = list(alpha.ss = c(1,1,1,1), alpha.sc = c(0,0,0,0)),

algorithm = "port")

summary(salix.model4r1)

######teste model removing lambda######

anova(salix.model4,salix.model4r1)

Qsr1 = -2 * (logLik(salix.model4r1) - logLik(salix.model4))

df.Qsr1 = df.residual(salix.model4r1) - df.residual(salix.model4)

1 - pchisq(Qsr1, df.Qsr1)

######model reduced by removing alpha.ss######

salix.model4r2 = nls(formula=Nst1 ~ Nst0*(lambda[senv.fac]*exp(-(alpha.sc[senv.fac]*log(Nct0)))),

data=s.cov,

start = list(lambda = c(5,5,5,5),alpha.sc = c(0,0,0,0)),

algorithm = "port")

summary(salix.model4r2)

######tested model removing alpha.ss######

anova(salix.model4,salix.model4r2)

Qsr2 = -2 * (logLik(salix.model4r2) - logLik(salix.model4))

df.Qsr2 = df.residual(salix.model4r2) - df.residual(salix.model4)

1 - pchisq(Qsr2, df.Qsr2)

######model reduced by removing alpha.sc######

salix.model4r3 = nls(formula=Nst1 ~ Nst0*(lambda[senv.fac]*exp((-alpha.ss[senv.fac]*log(Nst0)))),

data=s.cov,

start = list(lambda = c(5,5,5,5), alpha.ss = c(1,1,1,1)),

algorithm = "port")

summary(salix.model4r3)

######tested model removing alpha.sc######

anova(salix.model4,salix.model4r3)

Qsr3 = -2 * (logLik(salix.model4r3) - logLik(salix.model4))

df.Qsr3 = df.residual(salix.model4r3) - df.residual(salix.model4)

1 - pchisq(Qsr3, df.Qsr3)

#If the LRT p-value is less than your alpha level (usually 0.05 or 0.10),

#you conclude that the unconstrained 2-parameter model offers significantly

#better goodness-of-fit than the 1-parameter model for your sample data.

##################END OF LRT#######################

#calculate confidence intervals of parameters#

confint(carex.model4)

confint(salix.model4)

#Calculate equilibrium abundance based on model parameters#

########FULL EFFECT########

########################################

#ambient,grazed#

lambda.c = 0.625 #params specific for each envt

lambda.s = 0.355

alpha.cc = 0.763

alpha.ss = 0.791

alpha.cs = 0.045

alpha.sc = 0.207

#equilibrium solution

ln.Ns1 = (alpha.cc*(log(lambda.s)))/((alpha.ss*alpha.cc)-(alpha.sc*alpha.cs))

exp(ln.Ns1)

#final cover of Salix at equilibrium in ambient, grazed

ln.Nc1 = (alpha.ss*(log(lambda.c)))/((alpha.cc*alpha.ss)-(alpha.cs*alpha.sc))

exp(ln.Nc1)

#final cover of Carex at equilibrium in ambient, grazed

########################################

#ambient,ungrazed

lambda.c = 1.144

lambda.s = 0.534

alpha.cc = 0.534

alpha.ss = 0.740

alpha.cs = 0.023

alpha.sc = 0.126

ln.Ns1 = (alpha.cc*(log(lambda.s)))/((alpha.ss*alpha.cc)-(alpha.sc*alpha.cs))

exp(ln.Ns1)

#final cover of Salix at equilibrium in ambient, ungrazed

ln.Nc1 = (alpha.ss*(log(lambda.c)))/((alpha.cc*alpha.ss)-(alpha.cs*alpha.sc))

exp(ln.Nc1)

#final cover of Carex at equilibrium in ambient, ungrazed

########################################

#warm,grazed

lambda.c = 0.381

lambda.s = 0.772

alpha.cc = 1.087

alpha.ss = 0.464

alpha.cs = 0.097

alpha.sc = -0.005

ln.Ns1 = (alpha.cc*(log(lambda.s)))/((alpha.ss*alpha.cc)-(alpha.sc*alpha.cs))

exp(ln.Ns1)

#final cover of Salix at equilibrium in warm,grazed

ln.Nc1 = (alpha.ss*(log(lambda.c)))/((alpha.cc*alpha.ss)-(alpha.cs*alpha.sc))

exp(ln.Nc1)

#final cover of Carex at equilibrium in warm,grazed

########################################

#warm,ungrazed

lambda.c = 0.704

lambda.s = 0.934

alpha.cc = 1.087

alpha.ss = 0.578

alpha.cs = 0.162

alpha.sc = -0.089

ln.Ns1 = (alpha.cc*(log(lambda.s)))/((alpha.ss*alpha.cc)-(alpha.sc*alpha.cs))

exp(ln.Ns1)

#final cover of Salix at equilibrium in warm,ungrazed

ln.Nc1 = (alpha.ss*(log(lambda.c)))/((alpha.cc*alpha.ss)-(alpha.cs*alpha.sc))

exp(ln.Nc1)

#final cover of Carex at equilibrium in warm,ungrazed

**Supporting Information 2. Algebraic equilibrium solution to best fit model**

*Original Equations:*

$$\frac{N_{c,t+1}}{N_{c,t}}= \lambda_{c} e^{-\alpha_{cc}ln(N_{c,t})- \alpha_{cs}ln( N_{s,t})a_{cc}}$$

$$\frac{N_{s,t+1}}{N_{s,t}}= \lambda_{s} e^{-\alpha_{ss}ln(N_{s,t})- \alpha_{sc}ln( N_{c,t})a_{ss}}$$

*Rearranged:*

$$\ln\frac{1}{\lambda_{c}}= -a_{cc}\ln N_{c,t}- a_{cs}\ln N_{s,t} \Rightarrow\ln\lambda_{c}= a_{cc}\ln N_{c,t}+ a_{cs}\ln N_{s,t}$$

$$\ln\frac{1}{\lambda_{s}}= -a_{sc}\ln N_{c,t}- a_{ss}\ln N_{s,t} \Rightarrow\ln\lambda_{s}= a_{sc}\ln N_{c,t}+ a_{ss}\ln N_{s,t}$$

$$\ln N_{c,t}= \frac{1}{a_{cc}} (\ln\lambda_{c}- a_{cs}\ln N_{s,t} )$$

$$\ln N_{s,t}= \frac{1}{a_{ss}} \left( \ln\lambda_{s}- a_{sc}\ln N_{c,t} \right)$$

*Substitution:*

$$\ln N_{s,t}= \frac{1}{a_{ss}} \left( \ln\lambda_{s}- \frac{a_{sc}}{a_{cc}} \left( \ln\lambda_{c}-a_{cs}\ln N_{s,t} \right) \right)$$

$$\ln N_{s,t}\left( 1- \frac{a_{sc} a_{cs}}{a_{ss} a_{cc}} \right)= \frac{1}{a_{ss}} \ln\lambda_{s}$$

$$\ln N_{s,t}= \frac{\frac{1}{a_{ss}} \ln\lambda_{s}}{1- \frac{a_{sc} a_{cs}}{a_{ss} a_{cc}}}$$

*Solution:*

$$\ln N_{s,t}= \frac{a_{cc}\ln\lambda_{s}}{a_{ss} a_{cc}- a_{sc} a_{cs}}$$

*Therefore:*

$$\ln N_{c,t}= \frac{a_{ss}\ln\lambda_{c}}{a_{cc} a_{ss}- a_{cs} a_{sc}}$$

We compared our analytical solutions to simulation models and tested for sensitivity to initial conditions. In the simulations, we evaluated the outcome using our parameterized models for each treatment at a range of starting values (5-95% cover), over 20 time steps. We stopped at 20 time steps because models reached equilibrium after 5-10 time steps and equilibrium did not change beyond that, tested up to 100 time steps.
